# Draft Whole-Genome Sequence of *Bacillus sonorensis* Strain L12, a Source of Nonribosomal Lipopeptides

**DOI:** 10.1128/genomeA.00097-13

**Published:** 2013-03-28

**Authors:** David B. Adimpong, Kim I. Sørensen, Dennis S. Nielsen, Line Thorsen, Thomas B. Rasmussen, Patrick M. F. Derkx, Lene Jespersen

**Affiliations:** a University of Copenhagen, Faculty of Science, Department of Food Science, Frederiksberg C, Denmark; b Chr. Hansen A/S, Discovery Department, Hørsholm, Denmark; c Novo Nordisk, A/S, Department of Molecular Genetics, Måløv, Denmark

## Abstract

The *Bacillus sonorensis* L12 draft genome sequence is approximately 4,647,754 bp in size with a G+C content of 45.2%. Over 86% of the genome contains protein-encoding genes, including several gene clusters for *de novo* biosynthesis of the nonribosomal lipopeptides iturin, bacitracin, and fengycin, which could mean that the strain exhibits antifungal effects.

## GENOME ANNOUNCEMENT

*Bacillus sonorensis* is a Gram-positive aerobic-endospore-forming bacterium and a member of the *Bacillus subtilis* group of microorganisms. It was first isolated from the Sonoran Desert soil and is phenotypically and genotypically closely related to *Bacillus licheniformis* by sharing traits such as being facultatively anaerobic (1–3). However, the *B. sonorensis* and *B. licheniformis* species can be phenotypically distinguished based on colony pigmentation (1, 4) and sensitivity to different level of clindamycin (5). The *B. licheniformis* sp. is exploited industrially for large-scale production of many enzymes, antibiotics, and biochemicals (3) and works as a host for cloning of several genes encoding α-amylases, etc. Several members of the *B. subtilis* group are also used as probiotics and biocontrol agents in crop farming (6, 7). Thus, given that the industrially relevant organism *B. licheniformis* is closely related to *B. sonorensis*, about which little is known, we sequenced the genome of *B. sonorensis* L12, which was isolated from Gergoush primary starter materials in Sudan (4). It is expected that the information obtained can be used to explore the biotechnological relevance of this organism as well as its genomics and phylogenetic status related to members of the *B. subtilis* group of microorganisms and thereby enhance our limited knowledge on the *B. sonorensis* species.

The genomic DNA of strain L12 was isolated from overnight culture using a commercial DNA isolation kit (GenElute bacterial genomic DNA kit, NA2110; Sigma-Aldrich Co., St. Louis, MO). Sequencing was performed at the Beijing Genomics Institute (BGI, Shenzhen, China) using a combination of randomly sheared libraries with inserts of 0.5 to 2 kb in size. The genomic DNA was sequenced using the Illumina Hiseq platform with a total coverage of 70×. The reads were assembled into contigs using CLC Genomics Workbench v. 5 (CLC Bio, Aarhus, Denmark), which was also used to determine the nucleotide sequence statistics. Genome annotation and preparation for submission to GenBank were performed using the NCBI Prokaryotic Genomes Automatic Annotation Pipeline (PGAAP). Putative tRNAs were predicted using the tRNAscan-SE 1.21 Server (8) programs.

The assembled draft genome sequence of strain L12 contains 34 contigs, a genome size of 4,647,754 bp, and a G+C content of 45.2%. The genome was 425,108 bp larger than the *B. licheniformis* DSM13^T^ genome. Strain L12 has 78 tRNA genes and 4,236 protein-coding genes representing 86.8% of the draft genome sequence.

Preliminary genome analysis revealed that strain L12 carries gene clusters for *de novo* biosyntheses of the nonribosomal lipopeptides fengycin, iturin, and bacitracin, which have potential biotechnological applications (9, 10). It therefore represents a potential strain which can be used as a cloning tool for genetic engineering and production of improved and novel antimicrobial agents and also as a bioprotective agent for controlling plant fungal pathogens in crop farming. Further comparative genomic analyses will also provide valuable insight into the evolutionary and phylogenetic status of this species and contribute to a deeper understanding of its ecology and evolution.

### Nucleotide sequence accession numbers.

This whole genome shotgun project has been deposited at DDBJ/EMBL/GenBank under the accession number AOFM00000000. The version described in this paper is the first version, AOFM01000000.
